# From Ecology to Biotechnology, Study of the Defense Strategies of Algae and Halophytes (from Trapani Saltworks, NW Sicily) with a Focus on Antioxidants and Antimicrobial Properties

**DOI:** 10.3390/ijms20040881

**Published:** 2019-02-18

**Authors:** Concetta Maria Messina, Giuseppe Renda, Vincenzo Alessandro Laudicella, Rozenn Trepos, Marilyne Fauchon, Claire Hellio, Andrea Santulli

**Affiliations:** 1 Dipartimento di Scienze della terra e del Mare DiSTeM, Laboratorio di Biochimica Marina ed Ecotossicologia, Università degli Studi di Palermo, Via G. Barlotta 4, 91100 Trapani, Italy; concetta.messina@unipa.it (C.M.M.); giuseppe.renda02@unipa.it (G.R.); alessandro.laudicella@gmail.com (V.A.L.); andrea.santulli@unipa.it (A.S.); 2 Istituto di Biologia Marina, Consorzio Universitario della Provincia di Trapani, Via G. Barlotta 4, 91100 Trapani, Italy; 3 Biodimar, Laboratoire des Sciences de l’Environnement Marin (LEMAR), UMR 6539, UBO/IUEM, 29200 Brest, France; rozenn.trepos@googlemail.com (R.T.); marilyne.fauchon@univ-brest.fr (M.F.)

**Keywords:** polyphenols, defenses, anti-oxidants, antifouling, anti-microbial, *Cystoseira foeniculacea*, *Halocnemum strobilaceum*

## Abstract

This study aimed at the characterization of the antioxidant power of polyphenol extracts (PE) obtained from the algae *Cystoseira foeniculacea* (CYS) (Phaeophyta) and from the halophyte *Halocnemum strobilaceum* (HAL), growing in the solar saltworks of western Sicily (Italy), and at the evaluation of their anti-microfouling properties, in order to correlate these activities to defense strategies in extreme environmental conditions. The antioxidant properties were assessed in the PE based on the total antioxidant activity test and the reducing power test; the anti-microfouling properties of the two PE were evaluated by measuring the growth inhibition of marine fish and shellfish pathogen bacteria as well as marine surface fouling bacteria and microalgae exposed to the fractions. Similar polyphenol content (CYS 5.88 ± 0.75 and HAL 6.03 ± 0.25 mg gallic acid equivalents (GAE) g^−1^ dried weight, DW) and similar reducing power percentage (93.91 ± 4.34 and 90.03 ± 6.19) were recorded for both species, even if they exhibited a different total antioxidant power (measured by the percentage of inhibition of the radical 2,2 diphenyl-1-picrylhydrazyl DPPH), with CYS (79.30) more active than HAL (59.90). Both PE showed anti-microfouling properties, being inhibitors of adhesion and growth of marine fish and shellfish pathogen bacteria (*V. aestuarianus*, *V. carchariae*, *V. harveyi*, *P. elyakovii*, *H. aquamarina*) and fouling bacteria (*V. natriegens*, *V. proteolyticus*, *P. iirgensii*, *R. litoralis*) with minimum inhibitory concentrations comparable to the commercial antifouling products used as a positive control (SEA-NINE™ 211N). Only CYS was a significant inhibitor of the microalgae strains tested, being able to reduce *E. gayraliae* and *C. closterium* growth (MIC 10 µg·mL^−1^) and the adhesion of all three strains tested (*E. gayraliae*, *C. closterium* and *P. purpureum*), suggesting its promise for use as an antifouling (AF) product.

## 1. Introduction

Natural bioactive compounds have been the most successful source of potential drugs and, historically, are considered to have been the first drugs used by humans, with records dating back to Mesopotamia and Egypt more than 3000 years ago [1]. However, in the last two decades, major pharmaceutical companies have largely turned away from natural product (NP) discovery efforts due to difficulties in supply, screening, determination of mode of action, and characterization of NPs compared with synthetic libraries [2]. Nevertheless, NPs continue to provide unique structural diversity in comparison to standard combinatorial chemistry, presenting opportunities for discovering novel low molecular weight lead compounds and modes of action [1]. Nowadays, we are witnessing a renewed interest in NP discovery, due to the urgent need to discover, for example, new anti-microbials in order to fight the spread of bacteria strains resistant to conventional antibiotics [3,4], advances in analytical and isolation techniques [5], and a better understanding and exploration of the marine and coastal environments, which has resulted in the continuous discovery and isolation of thousands of unique new bioactive compounds yielded from marine organisms [1]. 

Marine ecosystems are indeed dynamic environments, and host organisms have adapted to live in such challenging habitats. This is especially true for sessile organisms, which employ numerous strategies to survive, ranging from spatial or temporal avoidance (ephemeral vs perennial species), to the production and secretion of physical and chemical barriers [6]. Marine plants, in response to stress factors, secrete secondary metabolites, which are accumulated in the cells and used as a deterrent against grazers, epiphytes, epibionts and other competitors [7,8], but which are also active in disease prevention [9] and protection from oxidative stress [10]. A unique variety of physical factors (salinity, dryness, elevate temperatures and high solar radiations) is found in hypersaline environments. These environments have been employed for human usages since ancient times, while nowadays, their biotechnological potential is being explored [11,12].

The artisanal production of sea salt in the Mediterranean area through solar evaporation of sea water in saltworks is an ancient technique established during the Phoenician and Roman periods [13]. Over centuries, sea salt production has been important to the economy of many Mediterranean coastal countries [14,15]. However, for the last 60 years, small saltworks and, more recently, also large mechanized saltworks have been faced with a continuous decline caused by the high costs of machinery, equipment and manpower [16]. 

Despite the decline experienced by salinas and saltworks, saltscapes are an invaluable source of cultural heritage, history, and biodiversity, with significant effects on tourism and the economy [17,18,19,20,21]. Thus, in order to improve the economic situation of saltworks, sea salt production is coupled with other activities that produce a financial return. Traditionally, the first storage basins, in which the salinity does not exceed 60‰, are employed for artisanal fisheries [22,23,24]. Saltworks, due to their extreme environmental conditions, are also employed for microorganism biomass production for aquaculture, cosmetic and biotechnological purposes [12,17,25]—processes which are, at the same time, linked to the quality of the salt produced [18]. Furthermore, great interest has been focused on the potential use of the plants which grow around saltworks [26]. Indeed, only a few species of plants are able to resist such challenging environments, and these belong to the halophytes group. Halophytes, unlike the vast majority of plants, evolved the ability to grow in salty soils (up to 70 g/L of dissolved solids for *Salicornia bigelovii* [27]). These plants have acquired the necessary features to successfully cope with salinity, drought, extreme temperature and luminosity. 

The aim of this study is the implementation and valorization of a traditional and ancient technique—the sea salt production in the Mediterranean—through the evaluation of the potential use of plants and algae developing near the saltworks, which may represent a valuable biomass to be employed in the future. Thus, we present the properties of aqueous polyphenol extracts (PE) from two organisms growing abundantly near the Mediterranean saltwork of Nubia (Trapani, Sicily): an intertidal brown alga *Cystoseira foeniculacea* L. (Grev. Emend. Sauvageau) and a halophyte *Halocnemum strobilaceum* (Pall.) Bieb 1819. *C. foeniculacea* is dominant on both sides of the outer edge of the solar saltwork, while *H. strobilaceum* develops in the pathways between the various ponds, in harsh and extreme conditions. *C. foeniculacea* is a common marine alga of the intertidal zone of the Sicilian coast; its tufted thallus grows to a maximum size of 90 cm, living at a depth of 50 cm [28]. The genus *Cystoseira* (Cystoseiraceae, Phaeophyta) contains about 50 species around the world [29], is widespread in Mediterranean and Atlantic intertidal zones (especially in temperate areas), and is relevant for its biotechnological use. *H. strobilaceum*, commonly known as “*salicornia strobilacea*”, is the only species of the genus *Halocnemum* that belongs to the family of Amaranthaceae. It is common in Asian and Mediterranean areas, where it forms small shrubs in soils characterized by high salt content [30]. In these areas, it can account from 24% to 43.4% of total plant cover [31]. It is commonly employed as livestock fodder due to its elevated protein content [32].

In this study, the bioactive properties of the two PE are screened for their polyphenol content, antioxidant activity, antimicrobial activity (through inhibition of adhesion and growth). Marine plants represent a unique source of NBCs [33]; studies on extract profiles of *Cystoseira* spp. evidenced their high phenolic content [34], including phlorotannins, halogenated phenolic compounds particular to the group of Phaeophyceae [35], while recent studies have highlighted the wide range of applicability of compounds retrieved from halophytes, from biofuel production [36] to medical and cosmetic purposes [37,38]. Despite the interesting bioactive properties demonstrated by extracts from halophytes, to our knowledge, few studies have covered the chemical composition and the bioactivity of *H. strobilaceum*. Extracts of *H. strobilaceum* are known to be a rich source of polyphenols and flavonoids like coumarins [39,40,41], which grant the extracts elevated antioxidant properties [42] and make *H. strobilaceum* an interesting source that should be deeply investigated. 

Furthermore, we evaluated the anti-microfouling properties of the two extracts. The space and substrate available are, along with the nutrients and light levels, among the most important limiting factors for the settlement and survival of a broad range micro- and macro-organisms (from bacteria and microalgae to macroalgae and invertebrates [43]). As a result, marine organisms have developed strategies for fast and efficient colonization of biotic and abiotic surfaces in the marine environment [6], a phenomenon defined as “biofouling”. Biofouling is ubiquitous in marine environments and represents a major nuisance for the shipping industry; the growth of organisms on ship hulls leads to increases in weight, frictional drag and fuel consumption, which account for more than 50M USD per year for the US navy alone [44]. Organism growth on artificial structures also leads to the erosion of the substrate due to modification of pH and the secretion of biofilm, causing damage to pillars, platforms and pipelines [45], and in the case of moving substrates, it can hasten the spreading of alien species [46]. In the past, in order to prevent settlement and development of organisms on man-made surfaces, antifouling (AF) coatings based on organotin compounds (such as TBT) were used [47,48]. However, the widespread use of toxic AF paints has resulted in high levels of environmental contamination and raised concerns about their effects on marine communities [49], with a massive toxic effect on non-target organisms and persistence in the environment [50,51,52]. Nowadays, the use of these type of AF paints have been banned worldwide, following an IMO (International Maritime Organization) decision in 2001 [53]. Therefore, research is being directed toward the development of new “environmentally friendly” coatings that should be efficient against adhesion and/or growth of fouling organisms, but that are also not persistent in the environment and are not toxic for non-target organisms [48]. 

Furthermore, with the goal of incentivizing efficient ecosystem-based approaches to aquaculture production in saltworks through integrated multitrophic aquaculture (IMTA), our research aims to underline the beneficial properties of some components of IMTA systems, such as marine algae and extremophyle plants, in order to provide valuable data for pilot studies. Halophytes, in particular, are becoming key players in the diversification and promotion of land-based IMTA [54]. 

## 2. Results

### 2.1. Polyphenol Content

**Figure 1:**
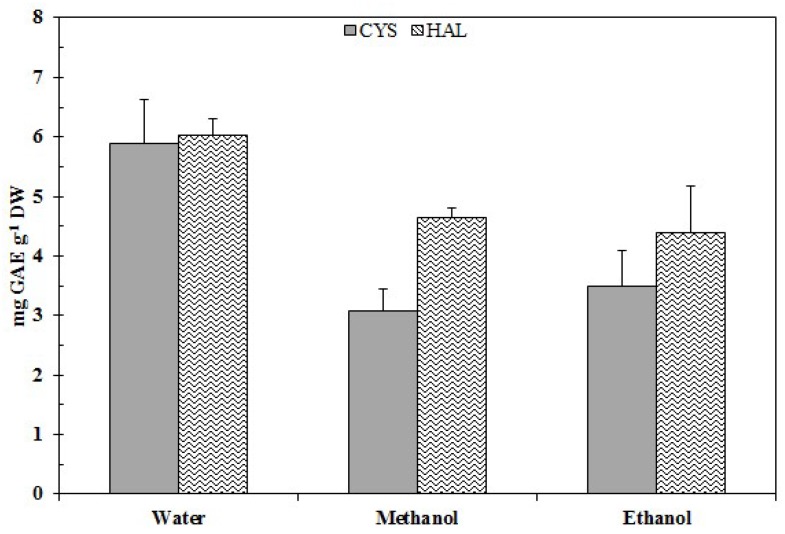
Total polyphenol content in the extracts obtained from CYS and HAL with different solvents.

The extraction of the total polyphenols was performed with different solvents, in order to evaluate the respective yield of polyphenols. The results, displayed Figure 1, evidenced that alcoholic extracts lead to the worst yields in total polyphenols; on the contrary, aqueous extracts lead to a better yield for both sources. The total polyphenol contents obtained were comparable in CYS and HAL, at 5.88 ± 0.75 mg gallic acid equivalent (GAE) g^−1^ of dry weight of samples (DW) for CYS and 6.03 ± 0.25 mg GAE g^−1^ DW for HAL. After the evaluation that the aqueous extract, PE led to the best yield; thus, it was used for the assessment of antioxidant and bioactive properties.

### 2.2. Antioxidant Properties

**Figure 2:**
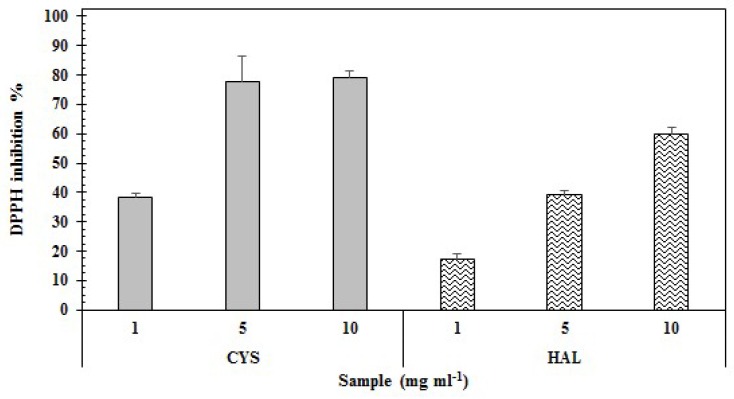
Percentage of inhibition of the radical DPPH in presence of different concentrations of aqueous extracts of CYS and HAL.

**Figure 3:**
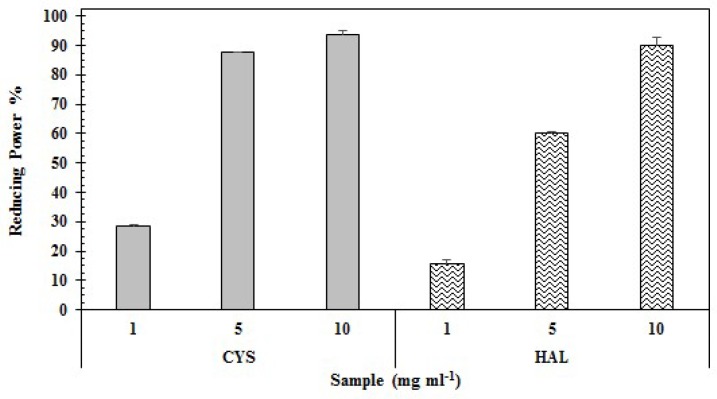
Reducing power (%) of CYS and HAL aqueous extracts at different concentrations.

The results of antioxidant power of the two PE are presented in Table 1 and detailed, with respect to the used concentrations for both tests, in Figure 2 and Figure 3.

Regarding the ability to scavenge the free radical DPPH, PE of CYS displayed a higher value for percentage of inhibition at the maximum concentration (79.30 ± 1.27%) compared to HAL (59.90 ± 6.74%) (Figure 2). These results were confirmed by the EC_50_ values: CYS, having the best antioxidant power, showed a lower EC_50_ (5.27 mg·mL^−1^) compared to the HAL samples (6.9 mg mL^−1^) (Table 1). 

As shown in Table 1, the reducing power obtained at the maximum concentration tested did not result in significantly different values between CYS and HAL (93.91 ± 4.34% and HAL 90.03 ± 6.19), even if the EC_50_ value was lower for CYS compared to HAL (3.59 vs 4.07 mg·mL^−1^). The higher activity of CYS (regarding reducing power) is shown in Figure 3.

### 2.3. Antibacterial and Anti-Microalgal Properties

Results of antibacterial and antimicroalgal assays are shown in Table 2. The results evidence the high antibacterial activity of CYS extract, especially against Vibrio strains. *V. aesturianus, V. carchariae, V. harveyi* and *V. natriegens* demonstrated high susceptibility to CYS extract (MIC values of 0.005 µg·mL^−1^). Although CYS were very efficient against all the other *Vibrio* strains tested, it did not inhibit the growth of *V. proteolyticus* (MIC > 10 µg·mL^−1^). Among the other strains tested, *P. irgensii* was highly inhibited by CYS (MIC 0.005 µg·mL^−1^), while *H. aquamarina*, *P. elyakovii*, *R. litoralis* and *S. putrefaciens* were not inhibited by the extract (MIC > 10 µg·mL^−1^). On the other hand, HAL demonstrated high antimicrobial properties against *H. aquamarina* (MIC < 0.0001 µg mL^−1^), *P. elyakovii* (MIC < 0.00001 µg·mL^−1^), *R. litoralis* (MIC < 0.0001 µg·mL^−1^), *V. aestuarianus* (MIC < 0.01 µg·mL^−1^) and *V. proteolyticus* (MIC < 0.01 µg mL^−1^), while the growth of other tested bacteria strains was not affected by the extract. The treatment with both extracts did not influence the adhesion of the bacterial strains assayed (MIC > 10 µg·mL^−1^).

The antimicroalgal assay results pointed out that the MIC values for growth inhibition were always higher than those obtained for the commercial product SEA-NINE™ 211N’: inhibition was produced by CYS on the growth of *E. gayraliae* and *C. closterium* (MIC 10 µg mL^−1^), while *P. purpureum* exhibited resistance to the extract (MIC > 10 µg·mL^−1^). Although HAL demonstrated interesting antimicrobial properties, it did not inhibit growth or adhesion abilities of the three microalgae strains used; on the other hand, CYS extract had a greater impact on the microalgae adhesion, as shown Table 2. All strains tested demonstrated an inhibition of adhesion following CYS treatment, with the most susceptible strains being, respectively, *E. gayraliae* (MIC 0.001 µg·mL^−1^) followed by *C. closterium* (MIC 1 µg·mL^−1^) and *P. purpureum* (MIC 10 µg·mL^−1^). 

## 3. Discussion

The field of biomimetics consists of the imitation of models, systems, and elements of nature for the purpose of solving complex human problems. In our study, we focus on the salt marsh environment, and especially on the investigation of defense strategies of two key species: *C. foeniculacea* and *H. strobilaceum*. We characterized the polyphenol fraction’s antioxidant power and defense potency against microorganisms in order to assess the potential of such halophytes, from saltmarshes, for economic valorization, as an untapped source of valuable secondary metabolites.

The two analyzed samples were characterized by similar polyphenol content in the aqueous extract (Figure 1). Phenolic compounds include a wide range of molecules, representing secondary metabolites in the plant kingdom, contributing to pigmentation, acting as antioxidants and protective agents against UV light, among other things [55]. Polyphenols in plants play many different roles: they can act as signal molecules in allelopathic plant-plant interactions and ripening of fruits, and also as defensive compounds [56]; they are active in the protection of the plant both from abiotic [37] and biotic factors, grazers [57], and wounds and diseases [56,58,59]. The anti-radical ability of phenolic compounds lies in their molecular structure, the availability of phenolic hydrogens, which can be donated to scavenge the radicals, and on the possibility of stabilization of the resultingly formed phenoxyl radicals [60].

Although quantitative determination of these compounds is hampered by their structural complexity and diversity, several methods can be used to determine polyphenols in plant extracts, among which the Folin-Ciocalteu method is the most utilized [61,62,63] and is also described in several pharmacopoeias [64,65]. In fact, this method measures the total concentration of phenolic hydroxyl groups in the plant extract which react with specific redox reagents (Folin-Ciocalteu reagent), thus forming a blue complex that can be quantified by visible-light spectrophotometry, using a reference substance [66]. The reaction generally provides accurate and specific data for several groups of phenolic compounds [67,68], and even if some studies have attempted to add some modifications, the methodology using the Folin-Ciocalteu is the preferred one, as it allows the obtaining of reliable results and an adequate comparison with the literature [61].

According to the chemical nature of polyphenols, the most common solvents employed for extraction are water and acetone, methanol or ethanol [69]. The choice of solvent has a significant effect on yield extraction. In our study, we chose to evaluate the bioactivity of polyphenols present in the water extract, as the preliminary test with different solvents showed that the best yield in polyphenols was obtained when using this solvent (Figure 1); this could represent a positive factor with respect to the utilization of these compounds, as the employment of water permits a broader range of application of the extracts, which would be narrowed by employing toxic organic solvents. In fact, to resolve this problem, many new techniques of extraction, such as supercritical fluid extraction (SFE), are being employed more and more in different industrial sectors, from food to pharmaceuticals [70].

Phaeophyceae and, in particular, the genus *Cystoseira* are well known to be relevant sources of polyphenols. In these organisms, polyphenols are usually localized in highly refractive and colorless vesicles called “physodes” [57]. Thanks to their elevated polyphenolic concentration, *Cystoseira* spp. extracts demonstrate antimicrobial [71,72], antifungal [73], antifouling [74], antioxidant and anti-inflammatory [29,75] properties. Our results indicate that CYS is characterized by 5.88 ± 0.75 mg GAE g^−1^ DW.

The results in the literature are very variable, ranging from high to low contents of polyphenols in this genus. In a paper by Chkhikvishvili and Ramazanov [76], the polyphenol content extracted by methanol was found to be equal to 21.6 mg g^−1^ DW, but this could vary with the time and location of collection and the methodology of extraction. To our knowledge, few studies have evaluated the polyphenol concentration in *Cystoseira* spp. aqueous fractions, and often the results are quite controversial. For example, Mhadhebi and coworkers evaluated the polyphenol content from aqueous extracts of *C. compressa* of Tunisian coast, finding a total polyphenol content of 61 ± 0.3 mg GAE g^−1^ DW [77]; on the same species from the Black Sea coasts of Turkey, Ozgun and Turan found a total polyphenol content of about 0.644 ± 0.1 mg g^−1^ DW [78]. The high variability of the results can be explained by the high degree of variability of this category of compounds, whose concentration is affected by a plethora of factors, such as the extraction conditions [69], seasonality [79], irradiance and depth [80], salinity [81], temperature, and others. Thus, for exploitation of polyphenols from the *Cystoseira* genus, it would be better to focus on controlled production in order to avoid yield variation.

Halophytes are another relevant source of polyphenols. In the Mediterranean area during the summer, the temperature may reach above 50 °C, and the salinity reaches the saturation point for NaCl (>5.1M NaCl) [11]. Survival in these extreme conditions is correlated with the generation of oxidative stress (e.g., large accumulation in the organism of reactive oxygen species “ROS”), which can be deleterious for cellular components, such as lipids, proteins and nucleic acids [82]. Under these conditions, usually, Halophytes develop powerful antioxidant systems employing several components, such as the increase of osmolyte biosynthesis (betains, proline, sugars or polyols), activation of enzymatic systems, and secretion and accumulation of bioactive substances such as polyphenols [83]. Thus, traditionally, there are many examples of halophytes being employed as a source for forage, human food, and animal feed thanks to their elevated protein levels, nutritional values and bioactive properties [84]. *H. strobilaceum* is a rich source of phenolic compounds, coumarins and flavonoids [39,40,41]; our results indicate a phenolic content for HAL of 6.03 ± 0.25 mg GAE g^−1^ DW (Figure 1), which is in line with our previous study [85], in which this extract was utilized to prevent the lipid peroxidation in fish fillets. Similar values were determined for aqueous extracts from others halophytes: *Limonastrium monopetalum* (2.6 mg GAE g^−1^ DW [37,86]), *Sueda maritima* (4.72 mg GAE g^−1^ DW) and *Sesuvium portulacastrum* (9.75 mg GAE g^−1^ DW [87]), *Mesembryathenum crystallinum* (1.43 mg GAE g^−1^ DW) and *Mesembryathenum nodiflorum* (1.72 mg GAE g^−1^ DW [88]), *Capparis spinosa* (30 mg GAE g^−1^ DW) and *Crithmum maritimum* (22 mg GAE g^−1^ DW; hot water extraction [89]). 

Phenolic compounds are, among the natural compounds, some of the most efficient ROS scavengers; thus, extracts usually demonstrate elevated levels of phenolic compounds and high antioxidant properties [90]. Aqueous extracts from *Phaeophyta* and halophytes, thanks to their rich phenolic content, are usually also granted elevated antioxidant properties. Recent studies have highlighted the antioxidant properties of extracts from *C. crinita*, *C. sedoides* and *C. compressa* [77], *Dyctiopteris membranacea* [91] and between halophytes *C. spinosa*, *C. maritimum* [89], *L. monopetalum* [37,86], *L. guyonianum* [86], *M. crystallinum* and *M. nodiflorum* [88]. Our findings reveal that both extracts have interesting antioxidant properties; however, CYS possessed a higher level of activity compared with HAL (DPPH EC_50_ 5.27 mg mL^−1^ and 6.9 mg mL^−1^, respectively; Table 1). Interestingly, a similar trend was highlighted for the reducing power of the two extracts, with CYS demonstrating stronger reducing power than HAL (Reducing Power EC_50_ 3.59 and 4.07 mg mL^−1^, respectively), (Table 1). The DPPH radical scavenging assay was used to evaluate the ability of the sample to scavenge the DPPH radical by donating one hydrogen molecule, thus indirectly measuring a sample’s reducing power [92]; meanwhile, via the reducing power assay, the reducing components of a sample can be directly evaluated by measuring the reduction of Fe^3+^ to Fe^2+^ [93,94]. In our case study, CYS demonstrated higher antioxidant properties than HAL (Table 1). Considering that we are evaluating two crude extracts, the reason for this may be related to numerous factors, such as differences in the phenolic compositions [95,96], presence of other molecules with antioxidant properties (as alginates, fucoidans and polysaccharides [97,98]), and/or the presence of halogenated phenolic substituents (for example, phlorotannins [99], which can lead to the higher antioxidant capacity of CYS). Further investigations are therefore necessary to give a more precise and certain explanation of this aspect.

Polyphenols, as stated above, are active substances in plant protection against pathogens, parasites, epiphytes, epibionts, grazers and other fouling organisms [100]. In our study, CYS and HAL resulted in efficient growth inhibition of most of the marine bacteria strains tested (Table 2). Among the bacteria strains tested, only *S. putrefaciens* appeared resistant to both extracts. Interestingly, the inhibition level, when it occurred, was higher than or of a similar magnitude to the one obtained by SEA-NINE™ 211N, which is, since the definitive ban of tin-based antifouling substances, one of the most active and widely used antifouling products on the market [101]. However, treatment with SEA-NINE™ 211N also blocked the attachment of the marine bacteria strains tested, while treatment with CYS and HAL did not produce any inhibition of the bacteria adhesion abilities (MIC > 10 µg mL^−1^; Table 2). CYS was particularly efficient in the inhibition of *V. aestuarianus*, *V. carchariae*, *V. harveyi*, *V. natrigens* and *P. irgensii*. *V. aestuarianus* and *V. harveyi*, which are pathogens for oysters and abalones [102], whereas *V. carchariae* is responsible for mass mortalities in groupers and other species of fish [103] and is considered to be a major nuisance for the aquaculture sector [104]. *V. natrigens* is non-pathogenic, but is ubiquitous in estuarine environments, and this strain is characterized by a really fast doubling rate, which, coupled with its biofilm producing ability and steel corrosion behavior, make it responsible for huge economic impacts on fisheries, fish farms and other human-made structures [105,106], while *P. irgensii* is a ubiquitous bacterium formerly isolated in the Artic [107]. Furthermore, CYS inhibited the growth of *E. gayraliae* and *C. closterium* (MIC 10 µg mL^−1^) and the attachment of all three of the strains tested (Table 2). Extracts from marine algae are known to be rich sources of antimicrobial compounds as a direct consequence of the environment in which they are living and the chemical ecology. The growth of epiphytes and epibionts on macroalgae can have numerous side effects, such as, for example: decreasing the light availability for the host, increasing weight and frictional drag, and decreasing the reproductive output [108]; thus, soft, settled or slow-moving marine organisms have developed strategies to keep their surfaces free of epibionts and epiphytes [109]. A famous example of this is *Delisea pulchra*, a Rhodophyta which produces halogenated enones and furanones able to inhibit quorum sensing-based communication between biofilm-forming bacteria and thus modulate their growth and virulence [110]. Phaeophyta extracts have been tested against marine bacteria [47,48,109,111,112,113,114,115], microalgae [116], macroalgae [117] and invertebrate larval settlements [112,113,114,118], and due to the elevated concentration of halogenated phenolics, terpenoids, glycolipids, glycerolipids, free fatty acids and chromanols, demonstrate elevated anti-microbial, anti-settling and antifouling properties [100]. 

On the other hand, HAL efficiently reduced *H. aquamarina*, *P. elyakovii*, *R. litoralis*, *V. aestuarianus* and *V. proteolyticus*, whereas no inhibition of growth or adhesion occurred on the microalgae strains tested (Table 2). *P. elyakovii* is a pathogen of macroalgae responsible for the spot-wounding disease in *Laminaria spp*. [119], *H. aquamarina* is pathogen for high economic value crustaceans such as lobsters [120], *V. proteolyticus* is a biofilm forming Vibrio species highly appreciated for the production and secretion of amminopeptidases [121], and *R. littoralis* is a common pigmented fouling bacterium [122,123]. Halophytes are another relevant source of bioactive compounds with interesting anti-microbial applications. In many countries, halophytes are part of the popular food tradition, and often their consumption is correlated with medical properties [124]. Studies have evaluated the anti-microbial properties of halophytes extracts, for example, compounds isolated from *Crithmum maritimum* strongly inhibited *Micrococcus luteus* and *Bacillus cereus* [125]; extracts from *Tamarix gallica* were active against both pathogenic bacteria (*Staphylococcus aureus*, *Staphylococcus epidermis*, *M. luteus*, *Escherichia coli*, *Pseudomonas aeruginosa*) and yeasts such as *Candida* spp. [124], while extracts from *Salicornia herbacea* exhibited efficient inhibition of multidrug-resistant bacteria strains [126]. Only a small number of studies have evaluated the antifouling properties of extracts from halophytes, mostly regarding extracts from mangrove species, evidencing interesting antifouling properties both against microfouling and macrofouling [127,128].

The biofouling process is divided into sequential stages (biochemical conditioning of the substrate, bacterial colonization, colonization of unicellular eukaryotes and colonization of multicellular eukaryotes [6]). The former stages include unicellular organisms (bacteria, protozoans and microalgae) which, via a conditioning process, allow settlement and enhance the colonization of other bigger fouling organisms [47]. Furthermore, microfoulers, while producing biofilm, release corrosive substances, which lead to the corrosion of the substrate and to a faster deterioration of the submerged structures [106]. So an interesting base for novel “environmentally friendly” AF coatings are compounds able to inhibit the first stages of fouling, and thus able to retard both the corrosion and the growth of macrofoulers on the substrate, while at the same time not being toxic or persistent in the aquatic environment [48]. For example, extracts from *Sargassum muticum* (Phaeophyta) inhibited the growth of the microalga *Fragilaria pinnata* without any additional toxic effects compared with other common commercial antifouling coatings and booster biocides [116]. A similar effect is observed from our results, CYS and HAL had a significant inhibitory effect on most of the bacteria strains tested, with MIC values closer or higher than those found for commercial AF products such as SEA-NINE™ 211N. Between the two extracts, CYS can be considered the most promising AF product due to its ability to inhibit both marine bacteria and microalgae. HAL was efficient in the inhibition of some of the marine bacteria tested; however, our results indicate an absence of inhibition of microalgae growth. This is in opposition to the findings of Deepa et al. [127], who assessed the effects of extracts from mangroves, highlighting their anti-microalgal activity against two marine diatoms as *Navicula subinflata* and *Nitzischia palea*. Nevertheless, these results cannot fully be compared if considering the taxonomical and the ecological differences between the sources of the extracts tested. As stated above, the antibacterial properties of halophytes were broadly evaluated, thanks also to their traditional use as medicinal herbs [124]. On the contrary, few studies have evaluated their potential as antifouling agents; for example, Kong et al. isolated several flavonoids from the halophyte *Apocynum venetum* that inhibited the growth of marine fouling bacteria such as *P. elyakovii*, *Bacillus thuringensis* and *Pseudomonas aeruginosa* [129]. 

Being a valuable source of antioxidants, *C. foeniculacea* and *H. strobilaceum* could also be considered, in the future, as organisms that can be part of saltwater-based IMTA systems. Focusing research on ecosystem-based approaches to aquaculture production will provide valuable data for the sustainable development of this economic sector. 

## 4. Material and Methods

### 4.1. Sample Collections and Processing

Three samplings of *Cystoseira foeniculacea* L. (Grev. Emend. Sauvageau) (CYS) and *Halocnemum strobilaceum* (Pall.) Bieb 1819 (HAL) were done, in duplicate, between May and July, close to the saltworks located on the Western Sicilian coast (Nubia, Trapani, Italy). Specimens of both organisms were kept in an herbarium and taxonomic identification was performed at the university of Western Brittany (France).

After collection, organisms were cleaned of epiphytes, rinsed with sterile distilled water, dried for 48 h at 40 °C, and then homogenized with a blender. Dried powder of CYS and HAL were submitted to extraction with different solvents (water, methanol and ethanol, ratio 1:10 *w/v*) in order to evaluate yields in total polyphenols. After evaluation, as the water extract led to the best yield in PE, we continued the assessment of antioxidant and anti-microbial properties using this fraction. In detail, to produce the water extract, the dried powders of both *C. foeniculacea* and *H. strobilaceum* were extracted with ultrapure distilled water (ratio 1:10 *w/v*; Simpak^®^2, Millipore, Italy following the methodology described by Akarpat et al. (2008) [130] for aqueous extraction of polyphenols, with minor modifications. After homogenization for 15 minutes at 40 °C, the solution was then sieved on 1 mm net and at 250 µm to remove coarse debris. The finest particles were removed by centrifugation (3000 rpm, 10 min, 4 °C) and the supernatant (containing the PE) was further filtered (Whatman 1001-270, pore size 11 μm) and stored at −80 °C for freeze-drying. 

### 4.2. Total Polyphenolic Content Evaluation

The determination of the total polyphenol content in CYS and HAL extracts was performed according to the Folin-Ciolcateau method [67]. Aliquots of extracts (50 µL) were diluted to 3 mL with ultrapure distilled water, to which was added 500 µL of ethanol (VWR chemicals, Radnor, PA, USA) and 250 µL of Folin’s reagent (VWR chemicals, Radnor, PA, USA). All the components were mixed and incubated for 5 min in darkness at room temperature. The reaction was started by adding 500 µL of 5% Na_2_CO_3_ to each tube; samples were again incubated for 1 h, in darkness, at room temperature and the absorbance was read at 725 nm with an UV/Vis spectrophotometer (Varian Cary 50 Scan, Palo Alto, CA, USA). The quantification was performed with reference to a standard curve of gallic acid (Sigma Aldrich, St. Louis, MS, USA) and the results expressed as mg of Gallic Acid Equivalents for each gram of dry weight of sample (mg GAE g^−1^ DW). Each sample was analyzed in triplicate.

### 4.3. Antioxidant Evaluation

The antioxidant properties of the polyphenolic extracts of CYS and HAL were determined by two methods: 1) the DPPH (2,2 diphenyl-1-picrylhydrazyl) radical scavenging assay, which aimed to evaluate the total antioxidant power; and 2) the reducing power test. 

#### 4.3.1. DPPH Radical-Scavenging Activity

The assay was performed according to the methods of Brand-Williams et al. [131] and Fukumoto and Mazza [132] in 96-well microplates (Nunc, Thermo Scientific, Waltham, MA, USA), and slightly modified according to Gharbi et al. and Manuguerra et al. [62,63]. Freeze-dried CYS and HAL samples were re-suspended in ultrapure distilled water and tested at concentrations ranging from 1 to 10 mg mL^−1^. 

Alpha-tocopherol and gallic acid were used as reference compounds (concentration 0.05–1 mg mL^−1^). Each measurement was performed in triplicate. The loaded plates were left to stand for 20 minutes in darkness at room temperature. The OD was recorded at 517 nm with an UV-Vis spectrophotometer (Infinite 200 Pro, Tecan, France). The scavenging effect was evaluated as follows: Scavenging activity = [1 − (Absorbance sample/Absorbance control)] × 100.

Where ODs, optical density of a sample, OD_b_, optical density of the blank and ODc, optical density of the control. The scavenging ability of the samples was also expressed as EC50 value, which is the effective concentration at which 50% of DPPH radicals were scavenged, and this was calculated via a linear regression relation.

#### 4.3.2. Reducing Power

The determination of the reducing power was performed according to Yen and Chen [94] and Kuda et al. [133], with minor modification [62,63]. Experiments were run in a 96-well microplate and extracts were used at concentrations ranging from 1 to 10 mg mL^−1^. The increase in absorbance is directly proportional to the reducing power of a sample; EC_50_ (Effective Concentration for 50% reduction) were determined for each extract.

### 4.4. Antimicrobial Activity

The effect of CYS and HAL polyphenol fractions on the adhesion and growth of bacteria and microalgae were evaluated at concentrations from 10^−4^ to 10 μg mL^−1^, as previously described by Trepos et al. [134], against the strains listed in Table 2. The marine organisms used for this assay were model organisms in antifouling studies [135]. All assays were run in 96-well plates. Methanol was used as a carrier solvent for CYS and HAL, and was evaporated before addition of microorganisms. All experiments were run in 6 replicates. SEA-NINE™ 211N (4,5-dichloro-2-*n*-octyl-4-isothiazolin-3-one; Sigma, France) was used as reference compound and assayed at final concentrations ranging from 0.01 to 10 μg mL^−1^.

#### 4.4.1. Antibacterial Assays

Bacterial strain adhesion and growth were determined according to the methods of Thabard et al. [113]. Bacterial suspensions (100 μL aliquots, 2 × 10^8^ colony forming units/mL) were aseptically added to the CYS and HAL fraction-containing microplate wells (1.10^−5^ to 10 μg mL^−1^), and the plates were incubated for 48 h at 26 °C before assessment of bioactivity. Bacterial growth was monitored spectroscopically at 630 nm and treatments were compared to a blank (containing media only consisting of 0.5% peptone (Oxoid Ltd., Basingstoke, UK) in sterile filtered (Whatman 1001-270, pore size 11 μm) natural seawater). The minimal inhibitory concentration (MIC) for bacterial growth was defined as the lowest concentration which results in a decrease in OD. Bacterial adhesion assay was conducted by the staining method (with aqueous crystal violet (100 μL, 0.3% *v*/*v*)) described in Trepos et al. [134]. The MIC for bacterial adhesion was defined as the lowest concentration of compound that, after 48-h incubation, produced a decrease of the OD at 595 nm. The strains were considered resistant if they showed growth or attachment at 10 µg mL^−1^.

#### 4.4.2. Antimicroalgal Assays

Microplates containing, HAL and CYS fractions in a range of concentrations were prepared from MeOH stock solutions, as previously described [134]. Microalgal stock solutions were prepared using the chlorophyll analysis methodology [134]. The pretreated microplate wells containing extracts were left to evaporate in order to remove any trace of methanol, and were then treated with 100 μL of the algal stock solutions (0.1 mg chlorophyll a/mL). The plates were then incubated for 5 days at 20 °C under constant light exposure (140 μmol m^−2^·s^−1^). Both microalgal adhesion and growth inhibition were measured using the method described by Trepos et al. [134]. Growth was determined by analysis of liberated chlorophyll a quantified fluorometrically. MIC value for algal growth was defined as the lowest concentration yielding a decrease in growth compared to the blank (containing media only). The MIC for adhesion was defined as the lowest compound concentration causing a reduction in optical density after removal of the non-attached cells.

### 4.5. Statistical Analysis

Data are reported as average ± standard error. The MIC was considered to be the first value significantly compared with the growth control in each plate. Statistical analysis was performed with STATISTICA 6.0 software (StatSoft, Maisons-Alfort, France). Significant differences were assessed through Student’s *t*-test, with differences being considered significant at *p* < 0.05. 

## 5. Conclusions

The results of this study reveal the potential of *Cystoseira foeniculacea* and *Halocnemum strobilaceum* as sources of polyphenols, along with their broad range of utilization. The two resources might be considered in future as a possible source of income for a sector, the solar salt production in salinas and saltworks, which is experiencing a long economic decline. The phenolic content of the two extracts confers interesting antioxidant properties, which might allow cosmetic or nutraceutical applications, and the two species could also be considered as components for IMTA to ensure a high level of biomass production in a controlled manner.

Moreover, anti-microbial properties reveal that both extracts inhibited the growth of marine bacteria. However, only CYS also affected microalgae growth, suggesting a more efficient use as antifouling products. Both extracts inhibited the growth of pathogenic strains for aquaculture species, suggesting another possible use as probiotics in aquaculture feed, thanks also to the combination of their antimicrobial and antioxidant properties [136].

## Figures and Tables

**Table 1:** Antioxidant properties of CYS and HAL aqueous extracts.

Antioxidant Properties		CYS	HAL
**DPPH**	**Inhibition (%)**	79.30 ± 1.27	59.90 ± 6.74
	**EC_50_ (mg·mL^−1^)**	5.27	6.9
**Reducing power**	**Reduction (%)**	93.91 ± 4.34	90.03 ± 6.19
	**EC_50_ (mg·mL^−1^)**	3.59	4.07

**Table 2:** Antibacterial and anti-microalgal properties of CYS and HAL aqueous extracts.

		MIC (µg·mL^−1^)					
Species	Code	Growth			Adhesion		
Marine Bacteria	ATCC ^1^	CYS	HAL	SEA-NINE™ 211N’	CYS	HAL	SEA-NINE™ 211N’
*Polaribacter irgensii*	700398	0.005	>10	1	>10	>10	0.1
*Halomonas aquamarina*	14400	>10	0.0001	0.1	>10	>10	<0.01
*Pseudoalteromonas elyakovii*	700519	>10	0.00001	0.1	>10	>10	<0.01
*Roseobacter litoralis*	49566	>10	0.0001	1	>10	>10	<0.01
*Shewanella putrefaciens*	8071	>10	>10	1	>10	>10	1
*Vibrio aesturianus*	35048	0.005	0.01	<0.01	>10	>10	1
*Vibrio carchariae*	35084	0.005	>10	<0.01	>10	>10	<0.01
*Vibrio harveyi*	700106	0.005	>10	1	>10	>10	1
*Vibrio natriegens*	14058	0.01	>10	1	>10	>10	<0.01
*Vibrio proteolyticus*	53559	>10	0.01	0.01	>10	>10	0.01
**Microalgae strains**	**Algobank Code**						
*Exanthemachrysis gayraliae*	AC 15	10	>10	<0.01	0.001	>10	<0.01
*Cylindrotheca closterium*	AC 170	10	>10	<0.01	1	>10	<0.01
*Porphyridium purpureum*	AC 122	>10	>10	<0.01	10	>10	<0.01

^1^ American Type Culture Collection.
